# Classification of Muscular Dystrophies from MR Images Improves Using the Swin Transformer Deep Learning Model

**DOI:** 10.3390/bioengineering11060580

**Published:** 2024-06-07

**Authors:** Alfonso Mastropietro, Nicola Casali, Maria Giovanna Taccogna, Maria Grazia D’Angelo, Giovanna Rizzo, Denis Peruzzo

**Affiliations:** 1 Istituto di Sistemi e Tecnologie Industriali Intelligenti per il Manifatturiero Avanzato, Consiglio Nazionale delle Ricerche, 20133 Milan, Italy; alfonso.mastropietro@cnr.it (A.M.); nicola.casali@polimi.it (N.C.); 2 Dipartimento di Elettronica, Informazione e Bioingegneria, Politecnico di Milano, 20133 Milan, Italy; 3 Istituto di Tecnologie Biomediche, Consiglio Nazionale delle Ricerche, 20054 Segrate, Milan, Italy; mariagiovanna.taccogna@itb.cnr.it; 4 Unit of Rehabilitation of Rare Diseases of the Central and Peripheral Nervous System, Scientific Institute IRCCS Eugenio Medea, 23842 Bosisio Parini, Lecco, Italy; grazia.dangelo@lanostrafamiglia.it; 5 Neuroimaging Unit, Scientific Institute IRCCS Eugenio Medea, 23842 Bosisio Parini, Lecco, Italy

**Keywords:** deep learning, classification, MRI, Swin Transformer, neuromuscular diseases, skeletal muscle

## Abstract

Muscular dystrophies present diagnostic challenges, requiring accurate classification for effective diagnosis and treatment. This study investigates the efficacy of deep learning methodologies in classifying these disorders using skeletal muscle MRI scans. Specifically, we assess the performance of the Swin Transformer (SwinT) architecture against traditional convolutional neural networks (CNNs) in distinguishing between healthy individuals, Becker muscular dystrophy (BMD), and limb–girdle muscular Dystrophy type 2 (LGMD2) patients. Moreover, 3T MRI scans from a retrospective dataset of 75 scans (from 54 subjects) were utilized, with multiparametric protocols capturing various MRI contrasts, including T1-weighted and Dixon sequences. The dataset included 17 scans from healthy volunteers, 27 from BMD patients, and 31 from LGMD2 patients. SwinT and CNNs were trained and validated using a subset of the dataset, with the performance evaluated based on accuracy and F-score. Results indicate the superior accuracy of SwinT (0.96), particularly when employing fat fraction (FF) images as input; it served as a valuable parameter for enhancing classification accuracy. Despite limitations, including a modest cohort size, this study provides valuable insights into the application of AI-driven approaches for precise neuromuscular disorder classification, with potential implications for improving patient care.

## 1. Introduction

Muscular dystrophies (MDs) are widespread muscular disorders that pose relevant clinical and diagnostic challenges. They are all characterized by progressive muscle tissue atrophy, with resulting muscle weakness significantly contributing to cardiopulmonary failure and heightened mortality risks [1,2]. Among the diverse MD subclasses, including limb–girdle muscular dystrophies (LGMDs) and dystrophinopathies such as Becker muscular dystrophy (BMD), some of them exhibit overlapping muscle involvement patterns [3], as observed in BMD and LGMD type 2 (LGMD2) [4,5].

Currently, the prevailing approaches for MD diagnosis and tissue characterization are muscle biopsy and genetic testing. Genetic assessment is the gold-standard method for a final diagnosis and it is usually based on genetic panels or next-generation sequencing (NGS) [6,7,8]. However, NGS, renowned for its capability to identify more than 90% of dystrophinopathy cases [9], is hindered by its high costs and complex operational demands, limiting its widespread application. Furthermore, NGS’s accuracy diminishes when the candidate gene fails to align with the phenotype, when disease-causing variants are elucidated by more than one gene, or when identified variants have unknown effects [10,11]. As for muscle biopsy, it can provide a quantitative characterization of the disease’s impact on the muscular tissues; however, it is an invasive procedure and offers only a local assessment of the sampling sites, making its accuracy dependent on the amount of collected samples [12].

In this context, magnetic resonance imaging (MRI) can have a promising role, being a non-invasive multiparametric tool for investigating skeletal muscle morphology and structure [13]. Widely integrated into clinical practice, MRI plays a pivotal role in shaping diagnostic hypotheses by unveiling distinct muscle involvement patterns, which is crucial for informed clinical decision-making [14]. Its versatility extends to the assessment of fatty infiltration and abnormal increases in muscle water content, making it indispensable for diagnosing myopathies. Multiparametric muscle MRI proves invaluable beyond mere diagnosis. It not only aids in pinpointing muscle groups with inflammatory changes, facilitating cost-effective biopsies, but also extends its utility to provide crucial insights into disease progression and treatment responses within clinical trials and practical medical settings [3,15,16,17]. This multifaceted approach becomes particularly advantageous when clinical presentations suggest the involvement of multiple myopathy groups when a diagnosis is evident but confirmatory tests remain inconclusive, or when genetic analyses reveal variants of uncertain significance [14]. Finally, MRI serves as a confirmatory tool for clinical evaluations and directs patients toward more complex diagnostic investigations, enhancing the overall diagnostic process.

Clinical reports based on MRI images rely heavily on the skills of individual experts, mainly radiologists and neurologists which make them partly subjective and potentially inaccurate. Recently, artificial intelligence (AI) methods have emerged as promising tools to support MR image analysis and improve the objectivity of the diagnosis. In particular, deep learning (DL) approaches can extract relevant features from medical images to classify specific patterns, enabling precise and unbiased diagnostic evaluations [18,19]. DL methods, and particularly convolutional neural networks (CNNs), have demonstrated effectiveness in clinical applications for various medical tasks [19]. Vision Transformer (ViT), a recent innovation in this field, can overcome traditional CNN performances [20]. ViT excels in capturing long-range dependencies through self-attention mechanisms, facilitating the analysis of relationships among distant elements in an image. Notably, Swin Transformer (SwinT) offers scalability, allowing it to process images of different sizes and adapt to diverse datasets and resolutions while maintaining competitive performance, reducing computational demands [21]. Impressively, SwinT approaches exhibit robust generalization capabilities across a spectrum of computer vision tasks with minimal task-specific adjustments, and their interpretability and mitigation of overfitting contribute to their appeal [22,23].

AI research for classifying neuromuscular disorders using MR images shows promise [24]. Previous studies used machine learning (ML) [25], DL [26,27], or both [28,29]. Some investigations distinguished MD from non-MD cases [26], others differentiated DMD from BMD [28,29], and one study classified DMD, congenital MD, and healthy controls [27]. To date, the most comprehensive model, based on the Mercuri score and data from 10 MDs, lacks automated MRI feature extraction [25].

Considering what has been described above, it is evident that the existing literature lacks research papers dedicated to the automatic detection and differential diagnosis among BMD, LGMD2, and healthy controls using a multi-class classification approach. This gap is especially significant due to the overlapping muscle involvement patterns observed in both BMD and LGMD2 cases. Additionally, there remains a lack of literature focusing on the application of SwinT in this specific domain.

To address the above-identified research gaps, this study aims to develop and test a novel three-class classification approach utilizing the Swin Transformer model to identify healthy individuals and patients affected by BMD and LGMD2. Furthermore, considering the intrinsic multiparametric nature of MRI protocols, our study aims to evaluate the best MRI contrast for classification performance. By undertaking this research, we anticipate contributing valuable insights that can enhance the accuracy and efficiency of muscular dystrophy diagnoses, ultimately improving patient care and outcomes.

## 2. Materials and Methods

### 2.1. Subjects

A total of 75 MRI scans (from 54 subjects) were included in this retrospective study. Among them, 36 scans come from 15 subjects who were acquired multiple times (up to 3 times) in a longitudinal study. More specifically, the whole dataset included 17 scans from healthy volunteers, 27 from BMD patients (17 subjects), and 31 from LGMD2 patients (10 LGMD2A and 10 LGMD2B patients). Table 1 lists the main demographic and clinical variables describing the subjects.

The diagnosis for all patients included in this study was determined through an evaluation that included clinical phenotyping, genetic testing, and muscle biopsies (specifically for those with LGMD2). Gene sequencing revealed a mutation in the dystrophin gene in patients with Becker muscular dystrophy. In cases of LGMD2A, two mutations were identified in the CAPN3 (calpain3) gene, along with a reduction in muscular calpain 3 as indicated by western blot analysis. For patients diagnosed with LGMD2B, two mutations were found in the DYSF (dysferlin) gene.

Patients with beta-sarcoglycan-related LGMD (n = 3), NDD LGMD (n = 1), ANO5-related LGMD (n = 1), gamma-sarcoglycan-related LGMD (n = 1), caveolin-3 LGMD (n = 1), Fukutin-related-protein LGMD (n = 2), and congenital myopathy (n = 2) were excluded due to the low number of occurrences. Furthermore, nine patients diagnosed in the early stages were also excluded, as they did not exhibit any clinical signs detectable in MRI scans.

This study adhered to ethical standards, with written informed consent being obtained from all participating individuals, thereby affirming their voluntary agreement to take part in the research. Furthermore, the local ethical committee (IRCCS Medea) provided its formal approval, endorsing the project’s adherence to ethical guidelines and ensuring the protection of participants’ rights and welfare.

### 2.2. MRI Protocol

The MRI protocol included two sequences, a T1W gradient echo sequence and a DIXON sequence. The T1W sequence provides 2D structural images that can be used to identify and characterize the size of the different muscle bundles. DIXON sequences [30] are used to characterize the fat infiltration in the muscle tissue and provide three difference contrast maps: water (W) based contrast, fat (F) based contrast, and the fat fraction (FF) map.

MR images were acquired using a Philips Achieva dStream 3T MR scanner (Best, The Netherlands) and the sequence setup is presented in Table 2. All sequences were acquired on one of the subject tights (dx or sx depending on the clinical/research query) and the field of view (FOV) upper limit was set to the middle of the femoral head.

### 2.3. Dataset

**Figure 1:**
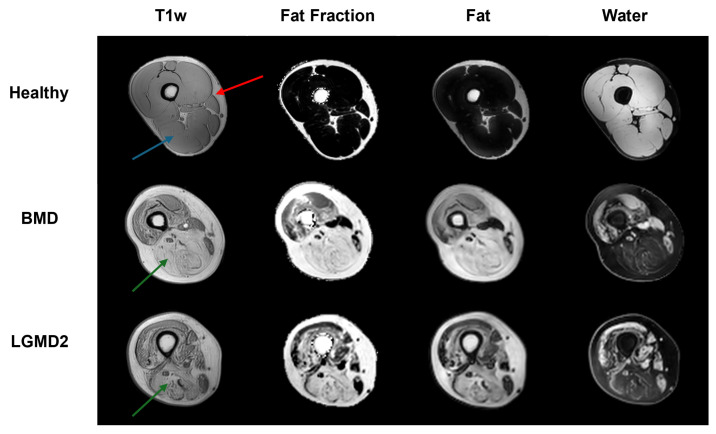
The image exemplifies the three distinct classes (healthy, BMD, and LGMD2), each delineated by unique image contrasts (T1w, FF, F, and W). The predicted subjects, chosen at random, show a typical cross-section of the thigh. The red arrow highlights the area of subcutaneous fat; the blue arrow (healthy subject) points to an example of intact skeletal muscle tissue, while the green arrows (BMD and GMD2 patients) show examples of heavily fat infiltrated muscles, a hallmark of neuromuscular disorders.

The dataset included a comprehensive collection of 75 MRI scans, each containing 30 slices, except for two scans of healthy control subjects that included 50 slices each, and one BMD scan consisting of 36 images. A subject-centric selection process was carried out to optimize the model training. For the classification experiments, a subset of 46 subjects, accounting for 67 scans and 2036 images, was selected for model training (60/61 scans) and validation (7/6 scans). To evaluate the effect of patients’ selection for model training on classification performances, 10 independent experiments were conducted, each time randomly shuffling the 67 scans ensuring that each of them was at least once included in the validation set. The test set included the remaining 8 subjects (8 scans; 260 images), which were randomly selected from the non-longitudinal dataset. An example of MR images included in the dataset is displayed in Figure 1.

### 2.4. Implementation of DL Architectures for the Classification Task

**Figure 2:**
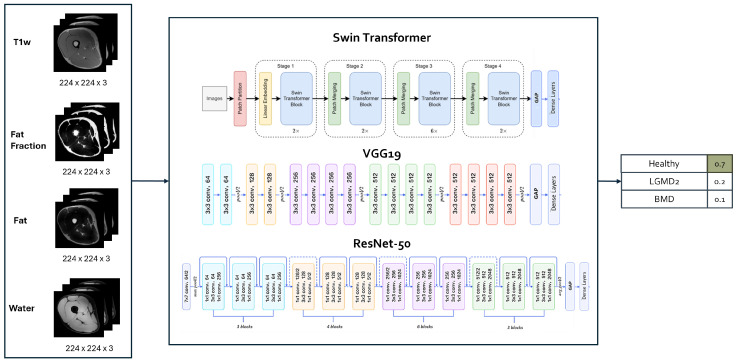
Architecture model selection. Figures adapted from [36,37,38].

As a first step, experiments involving various DL architectures were conducted with the specific aim of comparing CNNs and the SwinT. The considered architectures included (see Figure 2 for a graphical representation): (i) a 2D SwinT tiny [31] with a number of total trainable parameters = 27,914,621; (ii) a ResNet50 [32] with a number of total trainable parameters = 24,585,219; (iii) a VGG19 [33]: having a number of total trainable parameters = 20,288,579.

Considering the SwinT implementation, each patch was treated as a token with a size of 4, and its feature was formed by concatenating the raw pixel RGB values. Within each window, 7 patches were selected. In the tiny variant of the model, used in this work, the stages consist of 2, 2, 6, and 2 layers, respectively. The initial stage of the tiny variant has hidden layers with 96 channels. Each architecture employed was pre-trained on the ImageNet-1k [34] dataset and fine-tuned for three-class classification.

In all architectures, a global average pooling layer is placed at the top, followed by a dense layer with 512 neurons, a dropout layer with a dropout rate of 40% to mitigate overfitting, and a final dense layer with 3 neurons employing a softmax activation function for classification purposes. All images were resized to 224 × 224 pixels and background pixels were put to zero. To utilize the ImageNet-1k pre-trained architectures, the MRI single channel of the images was replicated across all three RGB channels of the ImageNet samples.

A data augmentation strategy was implemented to improve the generalizability of the proposed approaches. It included random (i) vertical flip; (ii) horizontal and vertical shifts, within a range of −10% to +10% of the total number of pixels in both directions; (iii) resizing of the image within 90% and 110% of the original size; (iv) rotations between −10 and +10 degrees. All experiments were performed using the Adam optimizer with a learning rate of 10^−5^, a batch size of 16, and a total of 40 epochs utilizing the Keras framework with a TensorFlow v. 2.10.1 backend on an NVIDIA GeForce RTX 3070 Ti GPU [35]. Independent training, validation, and testing of the models were conducted using the four available MRI contrasts: T1-w, FF, F, and W images.

### 2.5. Evaluation of the Classification Performance

The global performances of the classification task were assessed by means of accuracy (Acc), which represents the proportion of correctly classified slices out of the total slices in the dataset. Furthermore, to assess the class-specific performances, the F-Score, which represents the harmonic mean of precision and recall, was also calculated on the test dataset.

Formal definitions of Acc and F-score are, respectively, as follows:

(1) $$Acc=\frac{\sum_{n=1}^{N}TP+\sum_{n=1}^{N}TN}{\sum_{n=1}^{N}TP+\sum_{n=1}^{N}TN+\sum_{n=1}^{N}FP+\sum_{n=1}^{N}FN}$$

(2) $$F-score=\frac{2TP}{2TP+FP+FN}$$

where ‘N’ represents the total number of classes. ‘TP’ stands for True Positives, which are instances correctly identified as positive by the model. ‘FP’ denotes false positives, referring to instances incorrectly labeled as positive. Conversely, ‘TN’ refers to true negatives, which are instances correctly identified as negative, and ‘FN’ stands for false negatives, indicating instances that were incorrectly labeled as negative. The Acc and F-Score were computed as the average values obtained over 10 separate runs.

### 2.6. Statistical Analysis

The significance of the effects of the different DL architectures and contrasts on the classification task performances was assessed using a two-way ANOVA on rank-transformed data. Then, pairwise Wilcoxon tests with multiple testing corrections (false detection rate) were performed to compare groups of variables. First, the different DL architectures were compared. Then, the contrasts were compared among the implemented networks. *p*-values < 0.05 were considered significant.

## 3. Results

### 3.1. Global Classification Performances

**Figure 3:**
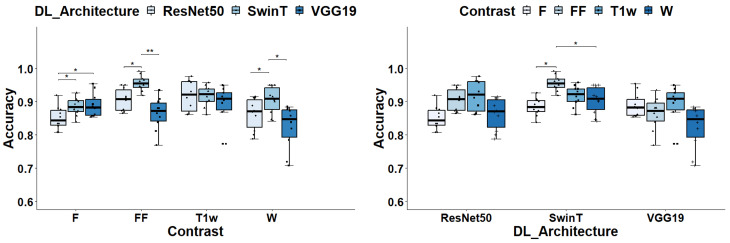
Global accuracy assessment for different DL architectures and image contrast. In the left panel, the box plots display ACC grouped by Image contrasts whereas in the right panel data are grouped by DL architecture. Significance markers indicate *p*-values, with * denoting *p* < 0.05 and ** signifying *p* < 0.01.

The selection of DL architectures and the type of image contrast play a crucial role in classification tasks, as evidenced by the results of the two-way ANOVA analysis. Notably, both factors—DL architecture and image contrast—had a significant influence on the Acc, with *p*-values registering at 1.97 × 10^−5^ and 2.04 × 10^−5^, respectively. When evaluating DL architectures against each other, grouped by image contrast types, SwinT provides the best median Acc scores. In detail, SwinT’s median Acc spanned from 0.885 for F images to 0.956 for FF maps, averaging 0.919. In comparison, VGG19’s median Acc varied from 0.848 with W images to 0.91 with T1w, averaging at 0.879; meanwhile, ResNet50’s median Acc fluctuated from 0.844 for F images to 0.921 for FF maps, with an overall average of 0.886. In general, SwinT performs significantly better or, at least, not worse than the other DL architecture in any contrast map. In particular, Swin-T has significantly better performances than both VGG19 and ResNet50 in the case of FF (*p* = 0.006 and *p* = 0.016, respectively) and W (*p* = 0.018 and *p* = 0.021, respectively) images, whereas SwinT is significantly better than ResNet50 in the case of F images (*p* = 0.048). When considering the T1w contrast, no significant differences among architectures were found. Please, refer to Figure 3 for further details.

In the evaluation of image contrasts across different deep learning architectures, SwinT stands out with FF image achieving a median Acc of 0.956, significantly outperforming its own performance with F and W contrasts, as evidenced by *p*-values of 0.027. Conversely, for VGG19 and ResNet50 architectures, T1-w images lead in performance with median Accs of 0.91 and 0.92 respectively. However, these differences did not reach statistical significance when compared to other contrasts.

### 3.2. Class-Specific Classification Performances

The analysis of class-specific classification performances confirms the critical influence of both the choice of DL architecture and image contrast on F-Scores. This is supported by the results of a two-way ANOVA analysis.

**Figure 4:**
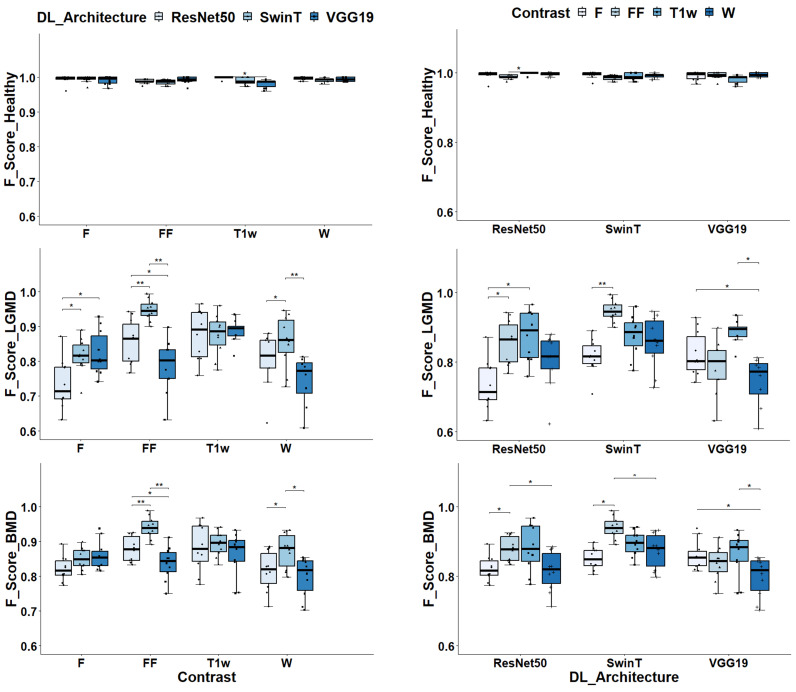
Class-specific F-Scores assessment for different dl architectures and image contrasts. In the left panels, the box plots display F-Scores grouped by Image contrasts and DL architectures in the case of healthy volunteers classification. In the middle panels, the same is displayed for LGMD classification whereas in the right panels, data are displayed for BMD classification task. Significance markers indicate *p*-values, with * denoting *p* < 0.05 and ** indicating *p* < 0. 01.

In particular, for the classification of healthy subjects, the DL architecture, and image contrast were found to have a significant impact, with *p*-values of 0.01 and 0.04, respectively. When classifying LGMD2 subjects, the *p*-values were lower, at 4.35 × 10^−6^ and 2.51 × 10^−6^, respectively. Similarly, for BMD classification, the *p*-values were 3.70 × 10^−5^ and 8.62 × 10^−6^. As to the classification of healthy subjects, as depicted in Figure 4, all DL models and contrast levels yielded high performance, with F-Scores ranging between 0.987 and 1. ResNet50 stood out with a median F-Score of 1. For VGG19, peak performance was achieved using F contrast, reaching a median F-Score of 0.994. Similarly, SwinT achieved its highest performance with F contrast, marked by a median F-Score of 0.997.

**Figure 5:**
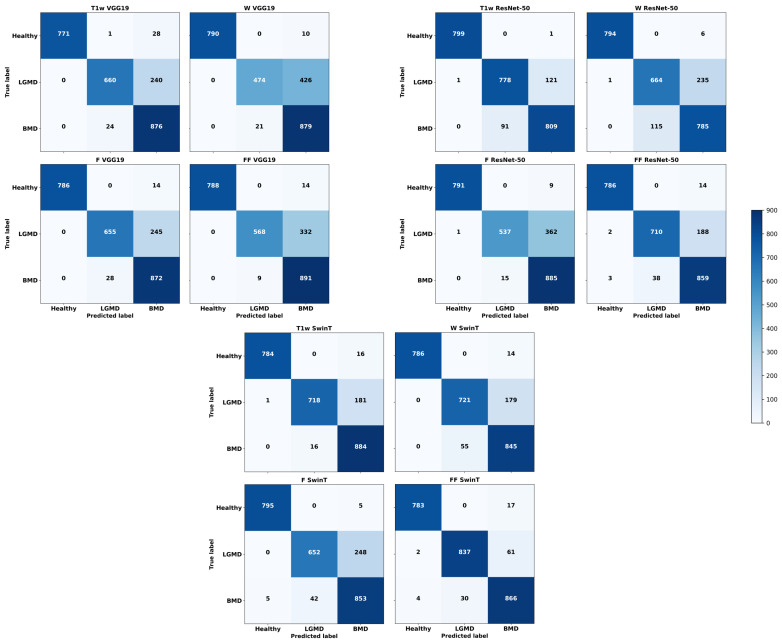
Cumulative confusion matrices reflecting various deep learning architectures and image contrast levels, derived from ten independent runs.

As depicted in Figure 5, ResNet50 exhibited remarkable accuracy, misclassifying a mere single image out of 800. In comparison, VGG19 had a slightly higher misclassification rate with 10 images out of 800–considering the cumulative results from 10 independent runs—specifically in the case of F. Even SwinT demonstrated high performance, incorrectly classifying 5 out of 800 images, even in the case of F. It is important to note that most of the images that were misclassified were predominantly identified as belonging to BMD.

In the classification of LGMD2 individuals, as shown in Figure 4, the median F-Scores ranged from 0.71 to 0.95 indicating the complexity of this task compared to classifying healthy volunteers. In particular, the SwinT model exhibited superior efficacy, achieving a median F-Score of 0.95. In contrast, VGG19’s optimal performance was observed with T1w images, attaining an F-Score of 0.88. Similarly, ResNet50 achieved the best median F-Score of 0.89 when utilizing T1w images.

Figure 5 can help elucidate how critical was the classification of LGMD2 patients. Notably, a significant number of misclassifications occurred during this task. Specifically, using T1w images, VGG19’s optimal setup incorrectly classified 240 out of 900 images, resulting in a 27% error rate. Similarly, ResNet50 had a 14% error rate, misclassifying 122 out of 900 T1w images. SwinT, utilizing FF images in its most effective configuration, had the lowest error rate at 7%, with 63 misclassifications out of 900 images. It is important to highlight that the majority of these errors involved mislabeling LGMD2 slices as BMD.

Finally, considering the classification of BMD patients, as shown in Figure 4, median F-Scores range from 0.82 to 0.94. Specifically, SwinT obtained the best performance using FF images with a median F-Score of 0.94. Conversely, both VGG19 and ResNet50 models reached their optimal performance with T1w images, each obtaining a median F-Score of 0.88. As illustrated in Figure 5, confusion matrices reveal that SwinT’s optimal setup resulted in 16 misclassifications out of 900 BMD images, equating to a 2% error rate. In comparison, VGG19 had 9 misclassifications, while ResNet50 had 15. It is important to note that the majority of these inaccuracies occurred when BMD images were incorrectly labeled as LGMD2.

### 3.3. Computational Times

Table 3 indicates that training times range from 11 to 22 min, with ResNet50 taking approximately 11 min, VGG19 taking about 22 min and SwinT taking approximately 17 min per run. Regarding inference times, they range from about 0.423 to 1.087 s on average.

## 4. Discussion

This study evaluated the SwinT model’s performance in tripartite classification tasks that differentiated between healthy individuals, BDM, and LGMD2 patients using muscle MRI scans. Additionally, the research aimed to determine the most effective MRI contrast for enhancing classification accuracy, considering the various protocols typical of clinical MRI settings. The use of DL methods for classifying neuromuscular diseases from MR images holds significant promise for enhancing clinical decision-making. This is particularly true in instances where there is substantial overlap in muscle involvement patterns, such as with BMD and LGMD2. In these conditions, muscles like the biceps femoris and semimembranosus are commonly and severely affected. Conversely, the sartorius and gracilis muscles are affected less often and with less severity [4,5]. Leveraging DL can aid in distinguishing these subtle variations, providing a more nuanced understanding that can inform treatment strategies.

In this paper, the SwinT method was evaluated against traditional convolutional neural networks (CNNs), namely VGG19 and ResNet50. These two architectures are extensively recognized for their effectiveness in classification tasks, as documented in the literature [39,40]. The results demonstrated that the SwinT, a cutting-edge deep learning model, delivered superior performance when compared to other convolutional neural network approaches when FF images are used as input. Notably, even when employing VGG19 and ResNet50 models, the overall accuracy (Acc) exceeded 0.90 in optimal configurations. This underlines the robust performance of CNNs, despite the superior results achieved with SwinT for this application. SwinT outperforms state-of-the-art techniques by effectively capturing long-range dependencies. This is achieved through its innovative self-attention mechanisms, which not only enhance the model’s scalability and generalization capabilities but also decrease computational requirements if compared to ViT [21].

The best contrast for use was found to be the FF maps, particularly when combined with the SwinT approach. FF maps allow the quantitative evaluation of fat infiltration in muscle tissue by combining F and W images, making them more informative than the originating images and even T1w images. FF maps serve as a critical tool for MD diagnosis by quantifying the percentage of fat infiltration in muscles, thereby enhancing diagnostic sensitivity and supporting the goal of early MD detection through MRI [41,42]. In addition to the insights gained from FF maps, it is noteworthy that T1w images have exhibited significant performance metrics. Specifically, they have achieved high Acc and F-scores, indicating superior contrast quality when utilized with both VGG19 and ResNet50 architectures. This suggests that T1w images may provide an optimal contrast for enhancing the performance of these CNN models.

When evaluating computational efficiency, the SwinT model necessitates about 17 min for each training session. This initial step, essentially performed only once, leads to a subsequent inference time of approximately 1.087 s, on average, per batch (i.e., 260 images). DL methodologies thus provide substantial time-saving benefits in their application (i.e., in the classification of new data). In contrast, visual image classification by a radiologist could take considerably longer than this automated process.

Previous studies have already explored the use of AI to classify different neuromuscular disorders from MR images showing interesting results. For example, in a study by Al-Wesabi and colleagues [28], the main goal was a binary classification to discriminate between BMD and non-BMD and between Duchenne muscular dystrophy (DMD) and non-DMD. Their approach integrated a DL capsule network (CapsNet) for feature extraction and employed the extreme learning machine (ELM) technique for classification, resulting in a diagnostic accuracy of 96.45%. Similarly, Gopalakrishnan and colleagues [29] introduced an innovative framework, based on the synergic DL method, for the automatic two-class classification between DMD vs. non-DMD as well as BMD vs. non-BMD, achieving classification performance rates of 96.18% for DMD and 94.25% for BMD, respectively. In another recent study by Yang et al. [26], an entirely deep learning-based method was developed, with a focus on enhancing binary classification accuracy across various types and subtypes of MD. The proposed approach based on a pre-trained ResNet50 model to distinguish dystrophinopathies from non-dystrophinopathies, yielded an accuracy of 91%. In Verdú-Díaz et al. [25], researchers employed a fatty replacement mercury score and a random forest-supervised machine learning technique to develop a model capable of accurately identifying correct diagnoses, achieving an impressive accuracy rate of 95.7%. Finally, Cai and colleagues [27] developed a three-class classification problem, distinguishing DMD, congenital muscular dystrophy (CMD), and normal subjects. They applied the ResNet18 for precise MD image classification and introduced an effective visualization method to highlight important image textures specific to DMD and CMD, visually different from each other, resulting in a classification accuracy of 91.7%.

Considering what was previously described, this research represents the first investigation into the application of the Swin Transformer (SwinT) architecture for a tripartite classification challenge using skeletal muscle MRI scans. When examining the quantitative outcomes, the SwinT, in conjunction with fat fraction (FF) images, achieved a peak accuracy rate of 95.6%. This benchmark places the methodology at the forefront of classification strategies for neuromuscular disorders, demonstrating its potential as a powerful diagnostic tool. This study acknowledges certain limitations. Primarily, the scope is narrowed by the modest cohort size and the finite selection of neuromuscular disorders examined. Additionally, the methodological approach of consolidating LGMD2A and LGMD2B into a single cohort, while potentially contentious, was employed as a means to strengthen the group’s statistical significance. These limitations should be taken into account when interpreting the findings and warrant consideration in future research activities.

## 5. Conclusions

In conclusion, this study marks a significant step forward in the application of DL methodologies for the classification of neuromuscular disorders using skeletal muscle MRI scans. Through the evaluation of the SwinT architecture alongside traditional CNNs, we have demonstrated the superior performance of SwinT in achieving high accuracy rates, particularly when utilizing FF images as input. The results underscore the potential of SwinT as a powerful diagnostic tool for enhancing clinical decision-making in the context of neuromuscular disorders, offering nuanced insights into muscle involvement patterns that can inform treatment strategies. Furthermore, our findings highlight the importance of selecting optimal MRI contrasts, with FF maps proving to be invaluable image-based biomarkers for enhancing classification accuracy. Despite certain limitations, this study lays the groundwork for future research studies aimed at further validating and expanding upon these findings. Overall, the outcomes of this investigation contribute to advancing our understanding of utilizing AI-driven approaches for the accurate classification of neuromuscular disorders, ultimately facilitating improved patient care and management.

## Figures and Tables

**Table 1:** List of demographic characteristics and clinical variables of the enrolled volunteers. MFM = Motor function measure; n.a. = not applicable.

	Total	Healthy Subjects	BMD Patients	LGMD2 Patients
N	75	17	27	31
Age (mean ± SD)	42.4 ± 11.8 yo	39.1 ± 11.5 yo	39.3 ± 10.3 yo	47.0 ± 11.9 yo
Gender (M/F)	50/25	10/7	27/0	13/18
Disease duration	25.7 ± 9.4 y (pat. only)	n.a	23.3 ± 8.3 yo	28.0 ± 10.1 yo
D1 (MFM) (mean ± SD)	17.3 ± 18.6 (pat. only)	n.a.	22.7 ± 18.6	12.9 ± 17.9
Ambulation (Y/N)	29/29 (pat. only)	n.a.	19/8	10/21

**Table 2:** Acquisition parameters for the MRI scans.

	T1W	DIXON
N° of slices	30/36/50 *	30/36/50 *
Slice thickness	6 mm	6 mm
In-plane voxel size	1 × 1 mm	1.6 × 1.6 mm
Acquired matrix	256 × 256	160 × 160
N° of echoes	1	12
Echo Time	2.025 ms	1.48–14.68 ms
Echo spacing	-	1.2 ms
Repetition Time	647 ms	16.11 ms
Flip Angle	90 deg	3 deg
Percentage sampling	100%	78%
Averages	2	2

* Two healthy subjects with 50 slices and one BMD patient with 36 images.

**Table 3:** Computational times.

Architecture	Training Time (min)	Classification Time (s)
ResNet50	11 ± 0.36	0.423 ± 0.014
SwinT	17 ± 0.08	1.087 ± 0.026
VGG19	22 ± 3.85	0.606 ± 0.006

Performance obtained using an NVIDIA GeForce RTX 3070 Ti GPU with 1.77 GHz Boost Clock and 8 GB RAM.

## Data Availability

The raw data supporting the conclusions of this article will be made available by the authors upon request.

## References

[1] Emery A.E. (2002). The muscular dystrophies. Lancet.

[2] Lovering R.M., Porter N.C., Bloch R.J. (2005). The muscular dystrophies: From genes to therapies. Phys. Ther..

[3] Nicolau S., Naddaf E. (2020). Muscle MRI for Neuromuscular Disorders. Pract. Neurol..

[4] Wattjes M.P., Kley R.A., Fischer D. (2010). Neuromuscular imaging in inherited muscle diseases. Eur. Radiol..

[5] Tasca G., Iannaccone E., Monforte M., Masciullo M., Bianco F., Laschena F., Ottaviani P., Pelliccioni M., Pane M., Mercuri E. (2012). Muscle MRI in Becker muscular dystrophy. Neuromuscul. Disord..

[6] Fanin M., Angelini C. (2016). Progress and challenges in diagnosis of dysferlinopathy. Muscle Nerve.

[7] Manzur A.Y., Muntoni F. (2009). Diagnosis and new treatments in muscular dystrophies. Postgrad. Med. J..

[8] Angelini C. (2020). LGMD. Identification, description and classification. Acta Myol..

[9] Okubo M., Minami N., Goto K., Goto Y., Noguchi S., Mitsuhashi S., Nishino I. (2016). Genetic diagnosis of Duchenne/Becker muscular dystrophy using next-generation sequencing: Validation analysis of DMD mutations. J. Hum. Genet..

[10] Nigro V., Savarese M. (2016). Next-generation sequencing approaches for the diagnosis of skeletal muscle disorders. Curr. Opin. Neurol..

[11] Ghaoui R., Cooper S.T., Lek M., Jones K., Corbett A., Reddel S.W., Needham M., Liang C., Waddell L.B., Nicholson G. (2015). Use of whole-exome sequencing for diagnosis of limb-girdle muscular dystrophy: Outcomes and lessons learned. JAMA Neurol..

[12] Joyce N.C., Oskarsson B., Jin L.W. (2012). Muscle biopsy evaluation in neuromuscular disorders. Phys. Med. Rehabil. Clin..

[13] Li K., Dortch R.D., Welch E.B., Bryant N.D., Buck A.K., Towse T.F., Gochberg D.F., Does M.D., Damon B.M., Park J.H. (2014). Multi-parametric MRI characterization of healthy human thigh muscles at 3.0 T–relaxation, magnetization transfer, fat/water, and diffusion tensor imaging. NMR Biomed..

[14] Venturelli N., Tordjman M., Ammar A., Chetrit A., Renault V., Carlier R.Y. (2023). Contribution of muscle MRI for diagnosis of myopathy. Rev. Neurol..

[15] Malartre S., Bachasson D., Mercy G., Sarkis E., Anquetil C., Benveniste O., Allenbach Y. (2021). MRI and muscle imaging for idiopathic inflammatory myopathies. Brain Pathol..

[16] Garibaldi M., Nicoletti T., Bucci E., Fionda L., Leonardi L., Morino S., Tufano L., Alfieri G., Lauletta A., Merlonghi G. (2022). Muscle magnetic resonance imaging in myotonic dystrophy type 1 (DM1): Refining muscle involvement and implications for clinical trials. Eur. J. Neurol..

[17] Forbes S.C., Willcocks R.J., Rooney W.D., Walter G.A., Vandenborne K. (2016). MRI quantifies neuromuscular disease progression. Lancet Neurol..

[18] Tobore I., Li J., Yuhang L., Al-Handarish Y., Kandwal A., Nie Z., Wang L. (2019). Deep learning intervention for health care challenges: Some biomedical domain considerations. JMIR MHealth UHealth.

[19] Kim M., Yun J., Cho Y., Shin K., Jang R., Bae H.j., Kim N. (2019). Deep learning in medical imaging. Neurospine.

[20] He K., Gan C., Li Z., Rekik I., Yin Z., Ji W., Gao Y., Wang Q., Zhang J., Shen D. (2023). Transformers in medical image analysis. Intell. Med..

[21] Liu Z., Lin Y., Cao Y., Hu H., Wei Y., Zhang Z., Lin S., Guo B. Swin transformer: Hierarchical vision transformer using shifted windows. Proceedings of the IEEE/CVF International Conference on Computer Vision.

[22] Wei C., Ren S., Guo K., Hu H., Liang J. (2023). High-resolution Swin transformer for automatic medical image segmentation. Sensors.

[23] Liu Z., Hu H., Lin Y., Yao Z., Xie Z., Wei Y., Ning J., Cao Y., Zhang Z., Dong L. Swin transformer v2: Scaling up capacity and resolution. Proceedings of the IEEE/CVF Conference on Computer Vision and Pattern Recognition.

[24] Piñeros-Fernández M.C. (2023). Artificial intelligence applications in the diagnosis of neuromuscular diseases: A narrative review. Cureus.

[25] Verdú-Díaz J., Alonso-Pérez J., Nuñez-Peralta C., Tasca G., Vissing J., Straub V., Fernández-Torrón R., Llauger J., Illa I., Díaz-Manera J. (2020). Accuracy of a machine learning muscle MRI-based tool for the diagnosis of muscular dystrophies. Neurology.

[26] Yang M., Zheng Y., Xie Z., Wang Z., Xiao J., Zhang J., Yuan Y. (2021). A deep learning model for diagnosing dystrophinopathies on thigh muscle MRI images. BMC Neurol..

[27] Cai J., Xing F., Batra A., Liu F., Walter G.A., Vandenborne K., Yang L. (2019). Texture analysis for muscular dystrophy classification in MRI with improved class activation mapping. Pattern Recognit..

[28] Al-Wesabi F.N., Obayya M., Hilal A.M., Castillo O., Gupta D., Khanna A. (2022). Multi-objective quantum tunicate swarm optimization with deep learning model for intelligent dystrophinopathies diagnosis. Soft Comput..

[29] Gopalakrishnan T., Sudhakaran P., Ramya K., Sathesh Kumar K., Al-Wesabi F.N., Alohali M.A., Hilal A.M. (2022). An Automated Deep Learning Based Muscular Dystrophy Detection and Classification Model. Comput. Mater. Contin..

[30] Eggers H., Börnert P. (2014). Chemical shift encoding-based water–fat separation methods. J. Magn. Reson. Imaging.

[31] Sayak P. (2022). Implementation of Swin Transformers in TensorFlow along with Converted Pre-Trained Models, Code for off-the-Shelf Classification and Fine-Tuning. https://github.com/sayakpaul/swin-transformers-tf.

[32] He K., Zhang X., Ren S., Sun J. Deep residual learning for image recognition. Proceedings of the IEEE Conference on Computer Vision and Pattern Recognition.

[33] Simonyan K., Zisserman A. (2014). Very deep convolutional networks for large-scale image recognition. arXiv.

[34] Deng J., Dong W., Socher R., Li L.J., Li K., Fei-Fei L. Imagenet: A large-scale hierarchical image database. Proceedings of the 2009 IEEE Conference on Computer Vision and Pattern Recognition.

[35] Chollet F. (2023). Keras. https://keras.io/.

[36] Tang Y. (2023). YanTang’s Blog. https://ehehe.cn/2023/08/09/Swin/.

[37] Rastogi A. (2022). ResNet50. https://blog.devgenius.io/resnet50-6b42934db431.

[38] Varshney P. (2020). VGG19. https://www.kaggle.com/code/blurredmachine/vggnet-16-architecture-a-complete-guide.

[39] Krishnapriya S., Karuna Y. (2023). Pre-trained deep learning models for brain MRI image classification. Front. Hum. Neurosci..

[40] Swati Z.N.K., Zhao Q., Kabir M., Ali F., Ali Z., Ahmed S., Lu J. (2019). Brain tumor classification for MR images using transfer learning and fine-tuning. Comput. Med Imaging Graph..

[41] Gaeta M., Messina S., Mileto A., Vita G.L., Ascenti G., Vinci S., Bottari A., Vita G., Settineri N., Bruschetta D. (2012). Muscle fat-fraction and mapping in Duchenne muscular dystrophy: Evaluation of disease distribution and correlation with clinical assessments: Preliminary experience. Skelet. Radiol..

[42] Morrow J.M., Sinclair C.D., Fischmann A., Machado P.M., Reilly M.M., Yousry T.A., Thornton J.S., Hanna M.G. (2016). MRI biomarker assessment of neuromuscular disease progression: A prospective observational cohort study. Lancet Neurol..

